# Circulating Type-1 Anti-Tumor CD4^+^ T Cells are Preferentially Pro-Apoptotic in Cancer Patients

**DOI:** 10.3389/fonc.2014.00266

**Published:** 2014-09-29

**Authors:** Amy K. Wesa, Maja Mandic, Jennifer L. Taylor, Stergios Moschos, John M. Kirkwood, William W. Kwok, James Harold Finke, Walter J. Storkus

**Affiliations:** ^1^Department of Dermatology, University of Pittsburgh, Pittsburgh, PA, USA; ^2^Department of Immunology, University of Pittsburgh, Pittsburgh, PA, USA; ^3^Department of Medicine, University of Pittsburgh, Pittsburgh, PA, USA; ^4^University of Pittsburgh Cancer Institute, Pittsburgh, PA, USA; ^5^Benaroya Research Institute at Virginia Mason University, Seattle, WA, USA; ^6^Department of Immunology, Cleveland Clinic Lerner Research Institute, Cleveland, OH, USA

**Keywords:** CD4^+^ T cells, peripheral blood, apoptosis, tumor, Type-1

## Abstract

Cancer patients frequently exhibit a deficiency in Type-1 (but not Type-2 or regulatory) CD4^+^ T cell responses against tumor-associated antigens (TAA), which may limit protection against disease progression or responsiveness to immunotherapy in these individuals. Since such deficiency was acutely evident in patients with active disease (AD), where chronic stimulation of anti-tumor CD4^+^ T cells would be expected and activation-induced cell death may be prevalent, we employed MHC Class II-peptide tetramers to characterize the frequency and apoptotic status of TAA- vs. influenza (FluM1) virus-specific CD4^+^ T cells in the peripheral blood of HLA-DR*0401^+^ patients with melanoma or renal cell carcinoma. We observed that Flu-specific CD4^+^ T cells ranged from 0.17 to 3.89%, while up to approximately 1% of CD4^+^ T cells reacted against individual TAA epitopes derived from the EphA2 or MAGE-6 proteins. The frequencies of EphA2 and MAGE-6-specific CD4^+^ T cells in patients were significantly correlated with AD and gender of the patient (i.e., females > males), while frequencies of Flu-specific CD4^+^ T cells were distributed within a normal range in all patients. Notably, patient CD4^+^ T cells reactive with MHC class II-TAA (but not MHC class II-Flu) tetramers were significantly enriched for a pro-apoptotic (Annexin-V^+^) phenotype, particularly amongst the Th1 (T-bet^+^) subset. These results suggest that the preferential sensitivity of TAA (but not viral)-specific CD4^+^ Th1 cells to apoptosis in melanoma patients with AD will need to be overcome for optimal clinical benefit of immunotherapeutic approaches to be realized.

## Introduction

Effective immunotherapy in the cancer setting is believed to be largely dependent upon the activation of endogenous CD8^+^ T effector cells that are capable of mediating sustained anti-tumor effector functions *in vivo* (1–7). Despite the ability of vaccines and immunotherapies to reproducibly augment circulating levels of CD8^+^ T cells reactive against tumor-associated antigens (TAA) in the peripheral blood of patients, rates for objective clinical responses in these trials have been disappointingly modest (8). These results support second-set deficiencies in the ability to effectively target and sustain circulating effector CD8^+^ T cells into/within sites of disease *in vivo* (9–11).

Type-1 CD4^+^ T (Th1) cells in particular appear crucial for optimal induction, recruitment, and long-term maintenance of therapeutic anti-tumor CD8^+^ T cells and anti-tumor immunity (12–15). Furthermore, CD4^+^ Th1-type helper cells are required for the reactivation and expansion of effector CD8^+^ T cells from memory precursors (16). Unfortunately, we and others have demonstrated that TAA (such as EphA2 and MAGE6)-specific, Th1 cell function is deficient in many cancer patients and that increased frequencies of TAA-reactive Th2- or Treg-type CD4^+^ T cells may be functionally dominant *in vivo* (17, 18), leading to a suppression of anti-tumor CD8^+^ T cell function (19, 20). The reason for biased Th1 dysfunction in cancer patients remains poorly understood.

Given reports that Type-1 CD4^+^ T cells are subjected to chronic antigen-stimulation making them differentially (vs. Type-2 or Treg) sensitive to activation-induced cell death (AICD) via an apoptotic mechanism (21), we hypothesized that TAA-specific CD4^+^ T cells freshly isolated from the peripheral blood of melanoma or renal cell carcinoma (RCC) patients with active disease (AD) would have a higher likelihood of exhibiting a pro-apoptotic phenotype. Based on our previous work supporting the common presence of anti-EphA2 and anti-MAGE6 CD4^+^ T cells in the peripheral blood of HLA-DR4^+^ patients with melanoma or RCC based on cytokine-based ELISPOT readout assays (17, 22), we now analyzed similar patients for the status of antigen-specific CD4^+^ T cells at the single cell level by flow cytometry by implementing fluorescently labeled MHC Class II-peptide tetramers. Our results suggest that patients with AD have greater frequencies of TAA-specific CD4^+^ T cells than patients rendered clinically free of disease, but that many of these antigen-specific T cells are actively undergoing apoptotic programing. These findings indicate that preferential death among TAA-specific CD4^+^ T cells likely contributes to anti-tumor immune dysfunction in patients and suggest that the therapeutic administration of stimuli to improve the survival and poly-functionality of anti-TAA Th1 cells may yield improved therapeutic benefit(s) to patients afflicted with cancers such as melanoma.

## Materials and Methods

### Isolation of PBMC and HLA typing

Peripheral blood was obtained by venipuncture from melanoma or RCC patients with their written consent under IRB-approved protocols. Peripheral blood mononuclear cells (PBMC) were isolated by density gradient separation (*d* < 1.077; endotoxin-free Histopaque, Sigma; St. Louis, MO, USA) and washed twice in phosphate buffered saline (PBS, pH 7.4; Life Technologies, Grand Island, NY, USA), then re-suspended in AIM-V medium (Invitrogen-Life Technologies, Carlsbad, CA, USA), and stored at room temperature (RT) overnight. HLA-DR4 positive donors were identified by flow cytometry using the 359-F10 mAb as previously described (23).

### CD4^+^ T cell isolation

CD4^+^ T cells were obtained from fresh PBMC using positive selection with MACS paramagnetic beads (Miltenyi; Auburn, CA, USA) on MiniMACS columns, according to manufacturer’s protocol and used for tetramer staining. In some cases, CD4^+^ T cells were isolated by negative selection (StemCell Technologies; Vancouver, BC, Canada), where indicated. Purity (>95%) was verified by flow cytometry after labeling isolated cells with CD3-FITC and CD4 PE (BD-PharMingen; San Diego, CA, USA).

### Detection of antigen-specific CD4^+^ T cells by flow cytometry

Peptide epitopes used in the construction of HLA-DR4-tetramers included: MAGE-6_121–144_, MAGE-6_246–263_, EphA2_53–68_, EphA2_63–75_, EphA2_663–677_, and inFluenza A matrix _60–73_ (FluM1) (17, 18, 24). Peptides were synthesized using FMOC chemistry by the University of Pittsburgh Cancer Institute’s (UPCI) peptide synthesis facility (shared resource). Peptides were >90% pure based on HPLC profile and MS/MS mass spectrometric analysis performed by the UPCI protein sequencing facility (shared resource). PE-labeled HLA-DR4-tetramers were constructed using these HLA-DR4 restricted epitopes as previously described (25). For detection of antigen-specific cells, CD4^+^ T cells (5 × 10^5^) were washed in FACScan buffer [0.2% BSA, 0.02% NaN_3_ (Sigma) in PBS] then incubated with human IgG (Sigma; 1 mg/ml in FACScan buffer) as a blocking reagent for 10 min. Cells were then incubated with PE-labeled HLA-DR4/peptide tetramers for 1 h at RT in the dark, then transferred for an additional 30 min incubation on ice in the presence of various mAb to surface markers [anti-CCR5-FITC, CD45RO-FITC, CD62L-CyChrome, CD25 FITC (BD-PharMingen), anti-CCR3-FITC (R & D Systems; Minneapolis, MN, USA)]. As a negative control, cells were stained in parallel with irrelevant isotype control mAbs (unconjugated or conjugated with FITC or CyChrome). Where noted, PBMC rather than isolated CD4^+^ T cells were used and anti-CD4-FITC (BD-Pharmingen) was used to identify CD4^+^ T lymphocytes co-binding PE-labeled HLA-DR4/peptide tetramers. After incubation(s) with mAb to surface antigens, cells were washed once in FACScan buffer and once in binding buffer [0.01 M Hepes (pH 7.4), 0.14 M NaCl, 2.5 mM CaCl_2_]. Apoptotic cells were detected by incubation with Annexin-V-biotin (BD-Pharmingen) for 15 min at RT, washed in binding buffer then incubated with Streptavidin (SA)-PerCP (BD-PharMingen) for 15 min at RT. Labeling of cells with PE-conjugated HLA-DR4/peptide tetramers followed by SA-PerCP (in the absence of annexin-V-biotin) did not result in SA-PerCP binding by tetramer^+^ cells as determined by flow cytometry, indicating that SA-PerCP (used to detect of annexin-V-biotin labeled apoptotic cells) did not bind to the biotin component of HLA-DR4-tetramers. Finally, cells were re-suspended in binding buffer (or FACScan buffer in instances where apoptosis was not being measured) prior to analysis on an EPICS-XL flow cytometer, with greater than 100,000 events were evaluated (Beckman-Coulter; Fullerton, CA, USA). For four-color flow cytometry, staining combinations included (1) anti-Tbet-FITC (Biolegend; San Diego, CA, USA), HLA-DR4/peptide tetramer-PE, annexin-V-biotin/SA-PerCP, with or without 7-AAD, (2) various FITC-mAb to surface markers combined with HLA-DR4 tetramer-PE and anti-CD62L-CyChrome. Combination cell surface marker and intracellular staining for T-bet was performed as previously described (26). Flow cytometry data were then analyzed using WinMDI software version 2.8.

### Statistical Analysis

SigmaStat software was used to analyze data. Statistically significant differences (*p* < 0.05) were determined using paired and un-paired *T*-tests.

## Results

### Quantitation of TAA-specific CD4^+^ T cell frequencies in the peripheral blood of patients with melanoma: correlation with disease status

**Figure 1:**
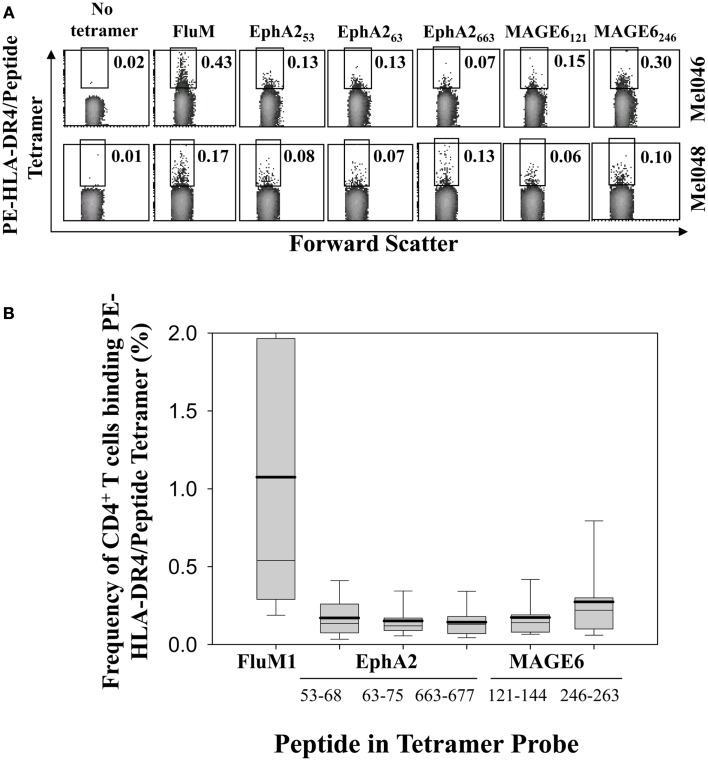
**Flow cytometric analysis of antigen-specific CD4^+^ T cells isolated from melanoma patients**. **(A)** Representative plots of flow cytometry analysis of HLA-DR*0401/peptide tetramer^+^CD4^+^ T cells from two melanoma patients (Mel046, Mel048). Live cells were gated based on forward and side scatter. Regions used to enumerate frequencies of (FluM1 or individual EphA2 or MAGE6 TAA) tetramer-reactive CD4^+^ T cells are shown. **(B)** Composite data obtained from flow cytometry analysis of CD4^+^ T cells using the indicated PE-labeled HLA-DR4/peptide tetramer probes in 15 HLA-DR*0401^+^ melanoma patients (Table 1; Mel031-Mel104). Box plots represent the range of responses for all patients, with mean frequencies shown as solid horizontal lines, and with whisker bars representing 5th and 95th percentiles for each HLA-DR4/peptide tetramer analyzed.

To determine the frequency of circulating anti-TAA Th cells in HLA-DR*0401^+^ melanoma patients, CD4^+^ T cells were freshly isolated from patient peripheral blood (Table 1) and incubated with PE-labeled HLA-DR4 tetramer complexes containing the MAGE-6_121–144_, MAGE-6_246–263_, EphA2_53–62_, EphA2_63–75_, EphA2_663–678_ TAA epitopes, or the (positive control) influenza A matrix (FluM1)_60–73_ viral epitope, then analyzed by flow cytometry (Figure 1). Frequencies of CD4^+^ T cells binding HLA-DR4/FluM1-tetramers in patients ranged from 0.17 to 3.89% (average 1.07 ± 1.07%, *n* = 15; Figure 1), with these levels significantly exceeding (*p* < 0.005) those noted for CD4^+^ T cells binding any given HLA-DR4/TAA peptide tetramer that ranged from 0.01 to 0.95% (average 0.18 ± 0.16%, *n* = 15). Isolation of CD4^+^ T cells by either positive or negative selection prior to tetramer analysis did not alter binding to HLA-DR4-tetramers, as the frequencies of tetramer^+^ cells detected within the isolated CD4^+^ T cell populations were equivalent to those detected in whole PBMC gated on CD4^+^ lymphocytes (data not shown).

**Table 1:** **Clinical characteristics of melanoma patients evaluated in this study**.

Melanoma patient no.	Age	Gender (M/F)	Disease stage	Disease status	Disease status at study conclusion	Previous treatment(s)
Mel031	53	M	II	NED	NED	S
Mel032	54	M	III	AD	NED	S, I
Mel036	48	F	II	NED	UN	S
Mel039	65	M	IV	AD	Deceased	S
Mel043	39	M	IV	AD	Deceased	S
Mel046	52	F	III	AD	AD	S
Mel048	32	F	III	AD	AD	S
Mel049	34	M	III	AD	Deceased	S, I, C
Mel060	45	M	IV	AD	Deceased	S
Mel073	52	M	IV	AD	NED	S, I
Mel093	46	M	IV	AD	Deceased	S, I, C
Mel094	57	F	II	AD	NED	S
Mel096	28	F	III	NED	NED	S
Mel103	66	F	IV	AD	NED	S, R, C
Mel104	67	F	IV	AD	Deceased	R
Mel612	38	M	IV	AD	Deceased	S, C
Mel633	56	M	II	NED	AD	S, I
Mel634	51	M	IV	AD	Deceased	S, I, C
Mel635	81	M	IV	AD	Deceased	S

*AD, active disease; C, chemotherapy; I, immunotherapy; NED, no evidence of disease; PBMC, peripheral blood mononuclear cells; R, radiotherapy; S, surgery; UN, unknown*.

**Figure 2:**
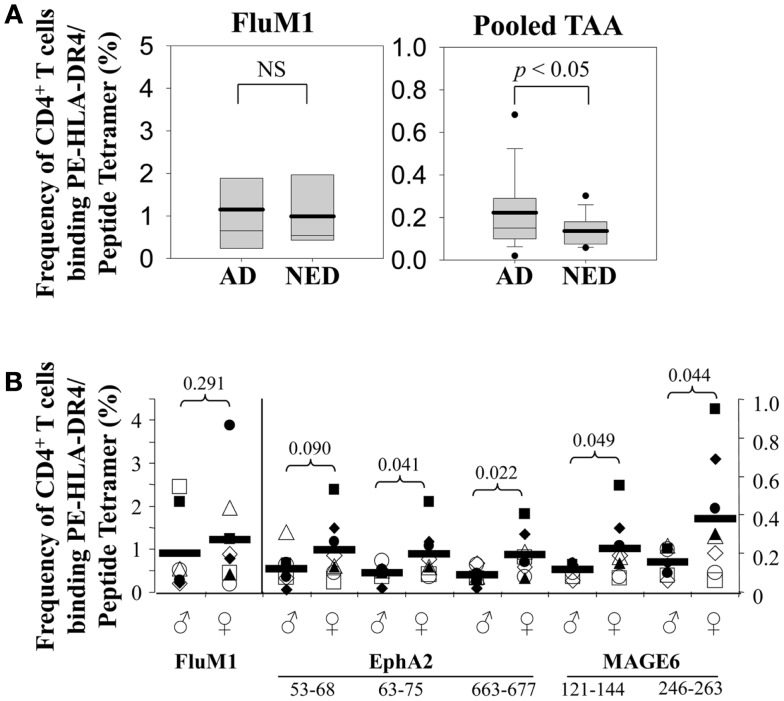
**Frequency of MAGE-6-, EphA2-, and Flu-specific CD4^+^ T cells in melanoma patients varies based on disease status and gender**. **(A)** Frequencies of antigen-specific CD4^+^ T cells in patients with active disease (AD; *n* = 8) vs. no evidence of disease (NED; *n* = 7) were determined using PE-labeled HLA-DR4/peptide tetramers and flow cytometry as described in Figure 1 and Section “Materials and Methods.” Composite data are depicted in Box plots. The means are represented as solid horizontal lines, whisker bars representing 5th and 95th percentiles, and outliers are represented by dots. Left graph represents FluM1-specific CD4^+^ T cells and the right graph represents pooled (three EphA2 epitopes + two MAGE6 epitopes) TAA-specific responses. **(B)** Frequencies of antigen-specific CD4^+^ T cells from seven male and eight female patients (no difference in age, disease stage, or presence/absence of disease) were determined using PE-labeled HLA-DR4/peptide tetramers and flow cytometry as indicated in Figure 1 and Section “Materials and Methods.” Symbols indicate data for individual patients, with mean frequencies indicated by heavy horizontal bars. *T*-test analysis was used to compare male vs. female patients, with *p*-values of differences between male/female cohorts indicated.

**Figure 3:**
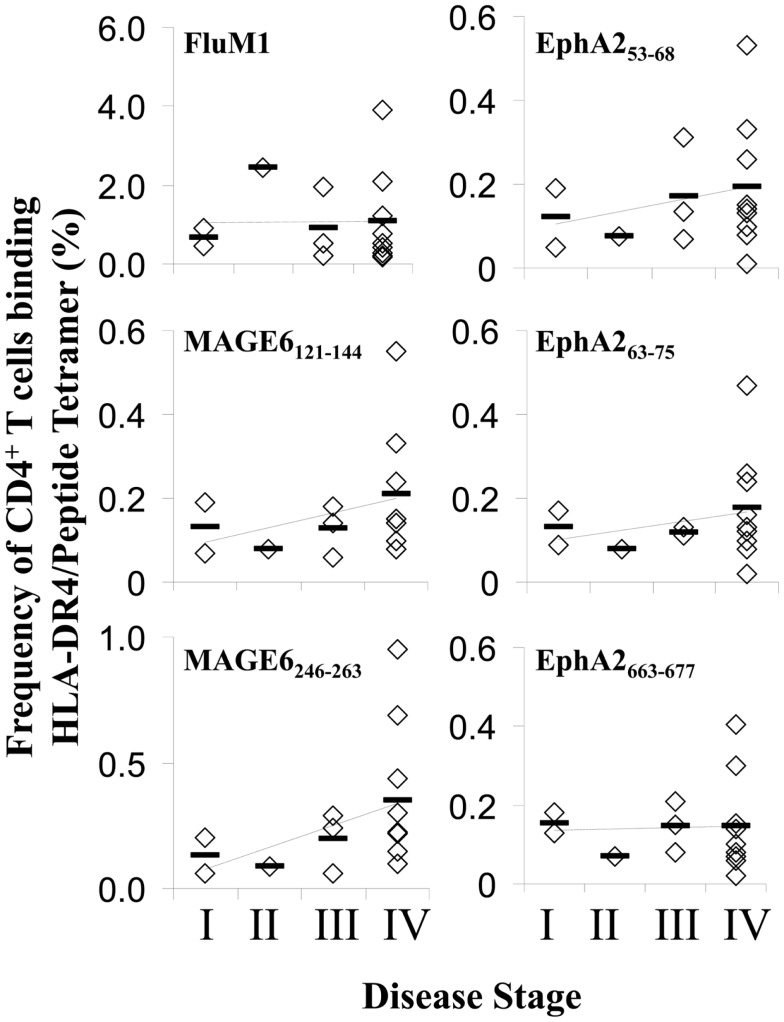
**Frequencies of antigen-specific CD4^+^ T cells in the peripheral blood of melanoma patients as a function of disease stage**. Frequencies of antigen-specific CD4^+^ T cells in melanoma patient PBMC was determined using PE-labeled HLA-DR4/peptide tetramers and flow cytometry as described in Figure 1. Patients were segregated based on clinical disease stage, with each diamond symbol representing data from a single patient. Heavy horizontal bars represent mean for each disease stage, with linear-regression trend lines overlaying each graph.

To determine whether the presence of melanoma affected the frequencies of TAA-specific CD4^+^ T cells in patients, we analyzed MAGE-6- or EphA2-reactive CD4^+^ T cells in individuals with no evidence of disease (NED) vs. patients with AD. While we observed no significant difference in the frequency of Flu-specific CD4^+^ T cells between these two patient populations (*p* = 0.39), we determined that frequencies of pooled TAA-specific CD4^+^ T cells in patients with AD were significantly increased when compared with patients who had been rendered NED as a consequence of prior therapy (*p* < 0.008; Figure 2A). Interestingly, TAA-specific CD4^+^ T cell frequencies in female patients were elevated (approximately twofold) when compared to male patients (*p* < 0.05), while Flu-specific CD4^+^ T cell frequencies were not significantly different between these patient cohorts (*p* = 0.29) (Figure 2B). Furthermore, we identified a trend for increased frequencies of TAA- (but not FluM1-) specific CD4^+^ T cells as a function of disease stage, although this did not reach statistical significance (Figure 3 and data not shown).

### Melanoma TAA-specific CD4^+^ T cells in patients are not biased with regard to naïve/memory/effector or regulatory phenotypes

To determine whether freshly isolated TAA-specific T cells in patients were of the naïve, memory, or effector phenotype, we assessed expression of the CD45RO and CD62L markers on tetramer^+^ CD4^+^ T cells. We observed that TAA epitope-specific CD4^+^ T cells were typically heterogeneous in nature, containing sub-populations corresponding to naïve (CD45RO^−^CD62L^+^), central memory (CD45RO^+^CD62L^+^), memory-effector (CD45RO^+^CD62L^−^), and effector (CD45RO^−^CD62L^−^) cells, with the patterns and subset distributions for TAA-specific CD4^+^ T cells similar to those obtained for patient-matched Flu-specific CD4^+^ T cells (Figure S1 in Supplementary Material). To determine whether TAA-specific T cells bearing a Treg phenotype were present in the peripheral circulation of melanoma patients; we analyzed tetramer^+^ CD4^+^ T cells for co-expression of CD25^hi+^. Among the patients evaluated, we determined that 5–15% of total CD4^+^ T cells were CD25^hi+^, regardless of “specificity” (i.e., HLA-DR4/FluM1-tetramer^+^ CD4^+^ T cells vs. HLA-DR4/TAA peptide tetramer^+^; data not shown). Thus, for a given patient, there was no apparent disease-associated bias in the naïve/effector/memory or regulatory-like phenotypes of TAA-specific CD4^+^ T cells.

### Circulating TAA (but not Flu)-specific CD4^+^ T cells are commonly pro-apoptotic in melanoma patients and enriched within the Type-1 (T-bet^+^) sub-population

**Figure 4:**
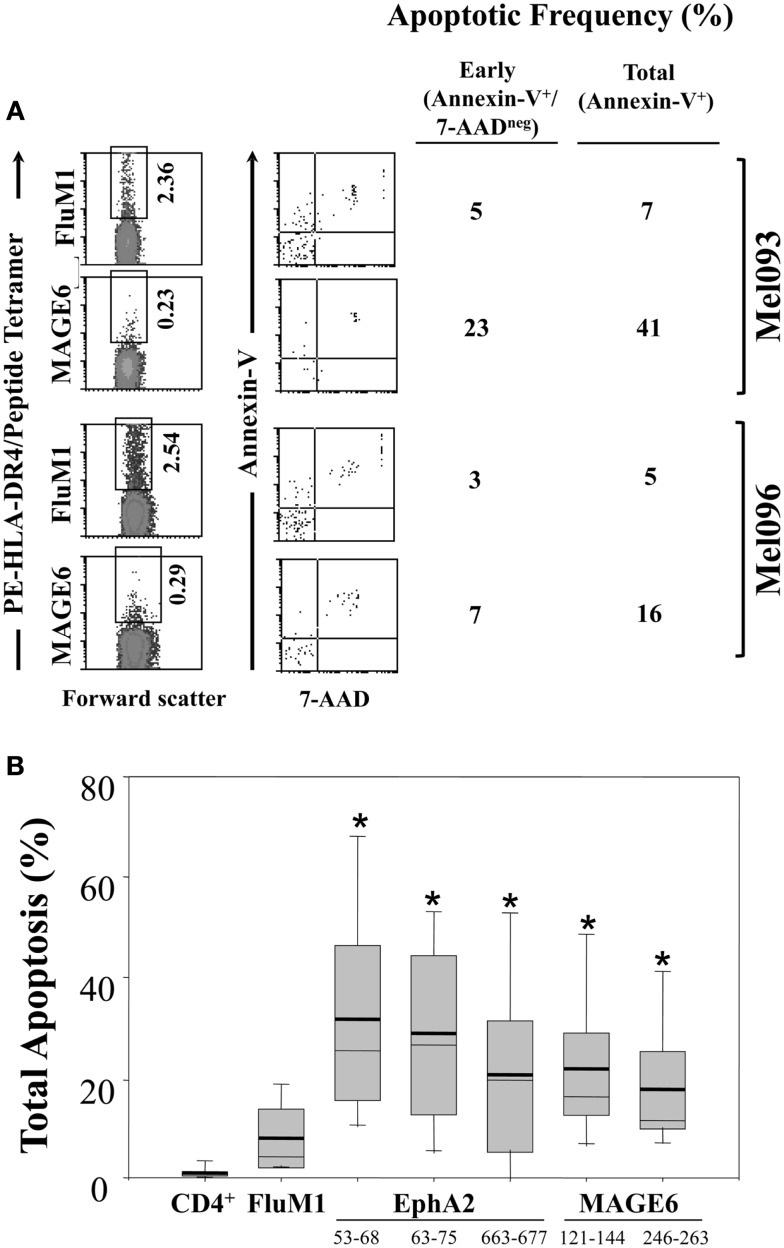
**Enhanced apoptosis of TAA (vs. FluM1)-specific CD4^+^ T cells in the peripheral blood of melanoma patients**. Flow cytometric analysis of CD4^+^ HLA-DR4/peptide tetramer^+^ T cells was performed as described in Figure 1. **(A)** Peripheral blood antigen-specific (tetramer^+^) CD4^+^ T cells from patients Mel093 and Mel096 were apoptotic status based on staining for Annexin-V and 7-AAD as monitored by multi-parameter flow cytometry (as described in Section “Materials and Methods”). Frequencies of early (Annexin-V^+^/7-AAD^neg^) vs. total (Annexin-V^+^ regardless of 7-AAD status) apoptotic events is tabulated. **(B)** Cumulative results for total CD4^+^ T cells and antigen-specific CD4^+^ T cells are depicted in Box plot format, with heavy horizontal lines representing the mean for each group, and whisker bars representing 5th and 95th percentiles (*n* = 13; i.e., Mel036-Mel104). *p < 0.05 vs. FluM1 (*T*-test).

**Figure 5:**
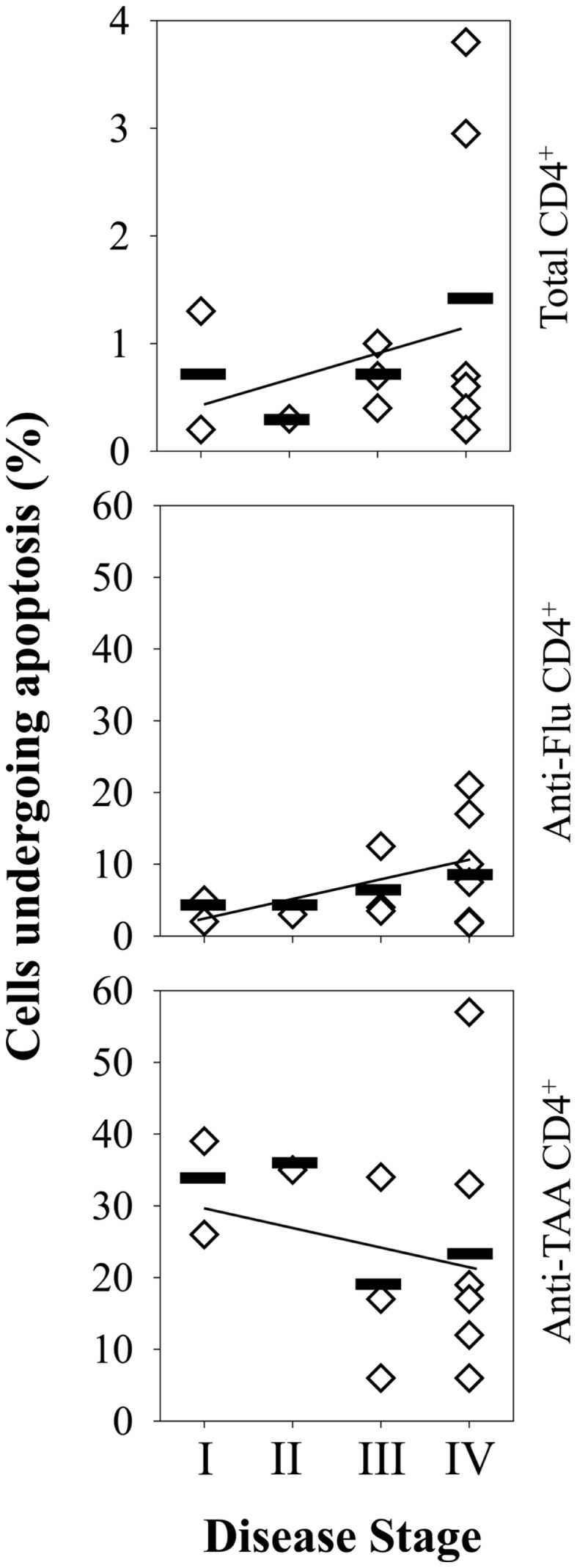
**TAA-specific CD4^+^ T cells in the peripheral blood of melanoma patients undergo enhanced rates of apoptosis (vs. Flu-M1-specific CD4^+^ T cells) at all stages of disease progression**. Frequencies of total apoptotic events among total CD4^+^ T cells and antigen-specific CD4^+^ T cells in melanoma patient PBMC (*n* = 13) were determined using PE-labeled HLA-DR4/peptide (pooled TAA or FluM1) tetramers and flow cytometry as described in Figure 4. Patients were segregated based on clinical disease stage, with each diamond symbol representing data from a single patient. Linear-regression trend lines overlay each graph.

Given our previous findings of functional deficiency in the Type-1 TAA-specific CD4^+^ T cell responses in melanoma and RCC patients (17, 18) and reports for the preferential sensitivity of Th1-type CD4^+^ T cells to AICD under conditions of chronic antigen-stimulation (27, 28), we next investigated whether HLA-DR4/TAA peptide tetramer^+^ CD4^+^ T cells in patients were prone to express an Annexin-V^+^ pro-apoptotic phenotype, and whether such phenotypes diverged from those associated with HLA-DR4/FluM1-tetramer^+^ CD4^+^ T cells (Figure 4A). While Annexin-V^+^ events were not common among total CD4^+^ T cells or HLA-DR4/FluM1-tetramer^+^ CD4^+^ T cells (i.e., 8.1 ± 6.6%) in a given patient, CD4^+^ T cells binding HLA-DR4/TAA peptide tetramers in patients displayed significantly elevated frequencies of Annexin-V^+^ sub-populations (*p* < 0.01 for each TAA epitope; *n* = 13; Figure 4B). Interestingly, the frequency of pro-apoptotic events among (FluM1- or TAA-) tetramer^+^ CD4^+^ T cells was not correlated with gender, age, or NED/AD status (data not shown) of patients. Although not reaching statistical significance, there was a trend for even FluM1- (in addition to TAA-) specific CD4^+^ T cells to exhibit elevated (i.e., >10%) frequencies of pro-apoptotic sub-populations in late-stage disease (Figure 5).

**Figure 6:**
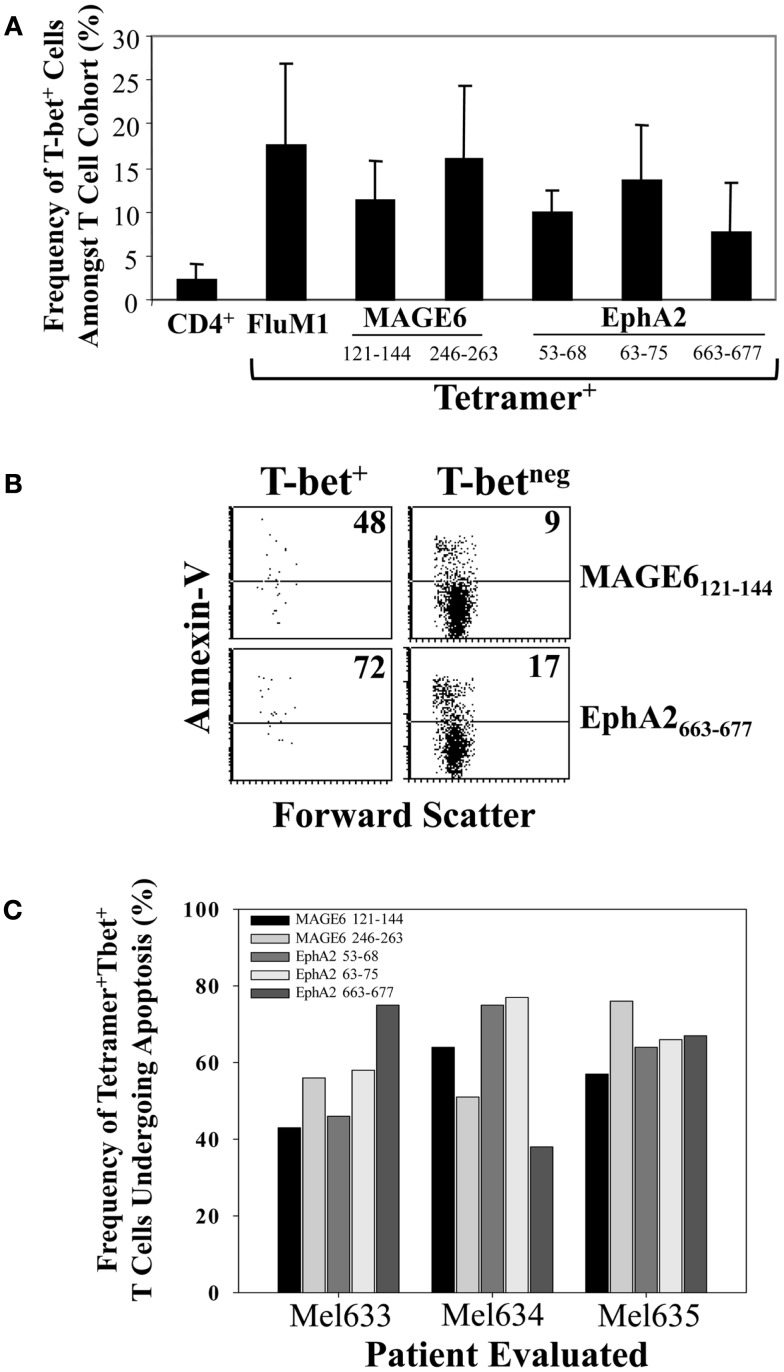
**The Type-1 subset of TAA-specific CD4^+^ T cells is a minority cohort that exhibits preferential sensitivity to apoptosis in the peripheral blood of melanoma patients**. Multi-parameter flow cytometry including intracellular staining for the Type-1 transcription factor T-bet (as described in Section “Materials and Methods”) was used to determine the frequency of Type-1 T cells among total CD4^+^ T cells or antigen-specific CD4^+^ T cell subsets [**(A)**; *n* = 4; i.e., Mel612, Mel633, Mel634, Mel635], the frequency of apoptotic (Annexin-V^+^) events among Type-1 (T-bet^+^) vs. non-Type-1 TAA-specific CD4^+^ T cells **(B,C)**. **(B)** Data derived from T cells obtained from patient Mel633.

To determine the frequency of antigen-specific CD4^+^ T cells that were Type-1 polarized and undergoing apoptosis, we performed multi-parameter flow cytometry including intracellular staining for T-bet, a transcription factor required for Th1 differentiation [(29); Figure 6]. We observed that TAA-specific Th1 cells were readily detectable in the circulation of melanoma patients, but that these cells represented a minority of total tetramer^+^ events in all cases (Figure 6A) and that these cells were preferentially undergoing apoptosis when compared with the T-bet^−^ subset of TAA-specific CD4^+^ T cells (Figure 6B). Indeed, for all TAA specificities evaluated, approximately 40–80% of the Th1 (T-bet^+^) sub-population exhibited a (pro)apoptotic phenotype (Annexin-V^+^; Figure 6C).

## Discussion

Based on our previous studies documenting Type-1 tumor-specific CD4^+^ T cell dysfunction in the peripheral blood of patients with melanoma or RCC (17, 18, 22), the current studies were developed to provide further insights into potential mechanisms that may underlie such deficiency in the advanced disease setting. Our past work utilized cytokine ELISPOT assays to enumerate and interrogate patient CD4^+^ T cell reactivity against specific HLA-DR4-presented epitopes derived from TAA such as EphA2 and MAGE-6. However, ELISPOT assays did not allow for multi-parameter assessment of epitope-specific CD4^+^ T cells to be pursued. Based on their ability to mark specific CD4^+^ T cells in multi-color flow cytometry assays, we developed HLA-DR4/peptide tetramers incorporating each of five previously defined TAA-derived epitopes: MAGE-6_121–144_, MAGE-6_246–263_, EphA2_53–62_, EphA2_63–75_, and EphA2_663–678_. These probes were then used to quantitate and provide additional phenotypic characteristics of circulating CD4^+^ T cells in HLA-DR4^+^ cancer patients.

We observed that TAA-specific CD4^+^ T cells were readily detectable in the peripheral blood of melanoma patients, regardless of whether these individuals had AD or if they were NED as a consequence of therapeutic intervention. Notably, the frequencies of TAA-specific CD4^+^ T cells were statistically elevated in patients with AD, whereas NED female patients on average displayed approximately twofold higher levels of TAA-specific Th cells when compared to male patients. Such tendencies were not observed for Flu-specific CD4^+^ T cell populations in these same patients. We believe that the former result may simply reflect the continued “booster” capacity of cross-presented TAA in patients with AD, which becomes limiting in patients characterized as NED (barring the presence of occult disease in these individuals). It remains unclear why higher frequencies of EphA2- and MAGE-6-specific CD4^+^ T cells were identified in female patients, although it has been previously reported that female melanoma patients have a generally better prognosis (i.e., longer survival) when compared to their male counterparts (30). It would be tempting to speculate that the presence of greater numbers of TAA-specific CD4^+^ T cells or the reported ability of CD4^+^ T cells from women vs. men to mount IFN-γ/IL-2-dominated recall responses to (pathogenic) restimulation (31) could serve as a foundation for understanding such clinical observations. The differential impact of reproductive hormones (estrogen enhancing vs. testosterone blunting) on antigen-specific T cell fate and function (32–34) must also clearly be considered within the context of our surprising findings. Additional analyses of a larger cohort of melanoma patients that have been immunologically interrogated in a longitudinal fashion to corroborate such a linkage would clearly be warranted.

**Figure 7:**
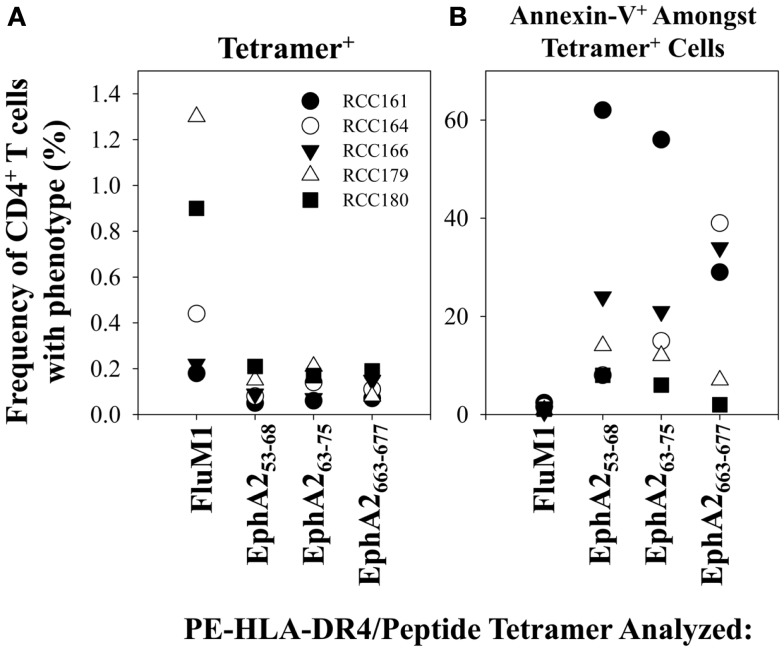
**TAA-specific CD4^+^ T cells in the peripheral blood of RCC patients undergo enhanced rates of apoptosis (vs. Flu-M1-specific CD4^+^ T cells)**. In **(A)**, frequencies of FluM1- and EphA2-specific CD4^+^ T cells in the PBMC of 5 HLA-DR4^+^ RCC patients with AD were determined using PE-labeled HLA-DR4/peptide tetramers and flow cytometry as described in Figure 1. In **(B)**, frequencies of total apoptotic events amongst total CD4^+^ T cells and antigen-specific CD4^+^ T cells in RCC patient PBMC (*n* = 5) were determined using PE-labeled HLA-DR4/peptide (pooled TAA or FluM1) tetramers and flow cytometry as described in Figure 4.

Since patients with AD are subjected to immunologic conditions of chronic antigenic stimulation, we believe it appropriate to consider general paradigms of eroding immune function in other clinically relevant models, such as chronic infectious diseases. In states of chronic viral infection, diminished Type-1 immunity (35) mediated via enhanced susceptibility of T cells to AICD (21, 28, 36) and/or the development of viral-specific Treg functional activity have been associated with the host’s inefficiency in clearing pathogens (37–39). Based on our current phenotypic analyses, we found little evidence for TAA-specific CD4^+^CD25^hi+^ T (Treg) cells, and indeed, no elevation in the frequency of this subset of T cells in the peripheral blood of patients vs. that reported for normal controls, consistent with a previous report for the bulk population of Treg (20). This leaves a likely possibility that the enhanced sensitivity of TAA-specific CD4^+^ T cells (especially the Th1-type) to AICD as a primary cause for our observation of Type-1 deficiency in melanoma patients with AD (17, 40). Our analyses in melanoma patients supported the elevated pro-apoptotic phenotype of TAA-specific (vs. FluM1-specific) CD4^+^ T cells based on Annexin-V^+^ staining in flow cytometry assays, which was clearly enriched within the Type-1 (T-bet^+^) sub-population of TAA-tetramer^+^ events across all tumor-associated antigenic specificities evaluated in this study. We have also obtained preliminary evidence for similar enrichment of Annexin-V^+^ events among CD4^+^ T cells reactive with TAA (but not FluM1) tetramer probes in HLA-DR4^+^ patients with RCC (Figure 7).

Whiteside’s group has previously reported that in patients with head-and-neck carcinoma or melanoma that circulating bulk CD8^+^ (but not CD4^+^) T cells frequently exhibit an early apoptotic phenotype (41–43), which may be related to an increased ratio of Bax/Bcl-2 or Bax/Bcl-xL expression within CD8^+^ T cells of patients (42). Our data would predict that a similar differential imbalance between pro (Bax)-/anti (Bcl-2, Bcl-xL, Mcl-1, Xiap)-apoptotic proteins is likely to occur within the subset of TAA-specific (but not bulk or viral-specific) CD4^+^ T cells in the peripheral blood of melanoma or RCC patients with AD.

In principle, such a clinical defect can be therapeutically corrected since alterations of pro- and anti-apoptotic molecules in lymphocytes may be modulated by multiple signals, provided it is via the cognate TCR, co-stimulatory/co-inhibitory molecules, as well as by cytokines. In particular, while *in vivo*-activated CD4^+^CD45RO^+^ T cells are susceptible to apoptosis, they can be “rescued” by cytokines or chemokines (44), including IL-2, IL-6, IL-7, IL-15, and CXCL12. Checkpoint inhibitors (anti-CTLA4, anti-PD-1/anti-PD-L1, anti-LAG-3, anti-BTLA), as well as agonists of CD40 or TLR, are also known to improve anti-tumor T cell survival (45–49). In addition, since tumor-derived exosomes serve as systemic instigators of T cell apoptosis (50), future interventional strategies may also be expected to improve the survival of anti-TAA Th1 cells in patients with cancer.

## Author Contributions

Author contributions included: study concept and design (Amy K. Wesa, Maja Mandic, Stergios Moschos, John M. Kirkwood, William W. Kwok, James Harold Finke, Walter J. Storkus), data acquisition (Amy K. Wesa, Maja Mandic), data analysis/interpretation (Amy K. Wesa, Maja Mandic, Jennifer L. Taylor, Stergios Moschos, John M. Kirkwood, William W. Kwok, James Harold Finke, Walter J. Storkus), drafting/editing of the manuscript (Amy K. Wesa, Maja Mandic, Jennifer L. Taylor, Stergios Moschos, John M. Kirkwood, William W. Kwok, James Harold Finke, Walter J. Storkus), approval of final content for journal submission and publication (Amy K. Wesa, Maja Mandic, Jennifer L. Taylor, Stergios Moschos, John M. Kirkwood, William W. Kwok, James Harold Finke, Walter J. Storkus).

## Conflict of Interest Statement

The authors declare that the research was conducted in the absence of any commercial or financial relationships that could be construed as a potential conflict of interest.

## Supplementary Material

The Supplementary Material for this article can be found online at http://www.frontiersin.org/Journal/10.3389/fonc.2014.00266/abstract
